# Web and/or MD?: Empirically testing the relationships between internet use and visits to healthcare professionals

**DOI:** 10.1016/j.socscimed.2025.118071

**Published:** 2025-04-15

**Authors:** Cayley Ryan-Claytor

**Affiliations:** Department of Sociology and Criminology, Pennsylvania State University, 601 Susan Welch Liberal Arts Building, University Park, PA, 16802, USA

**Keywords:** Internet, Online, Health information, Healthcare, Health services

## Abstract

The proliferation of the internet as a widely accessible repository of health information has sparked theoretical and empirical concerns about its potential use as a replacement for traditional healthcare services. Existing research highlights how use of the internet as a health information resource has influenced individuals’ experiences in healthcare settings, but has not yet explored its relationship with use of healthcare services. Using data from the National Health Interview Survey, I find a significant positive association between use of the internet to seek health information and visits to traditional healthcare providers. This association is not explained by factors related to respondents’ social and demographic characteristics, health status, or access to health services. This relationship is strongest among adults aged 18–39, suggesting that younger adults may be more inclined than their older counterparts to address health concerns using both the internet and traditional medical services. In line with Fundamental Cause Theory, the relationship is strongest among the highly educated, such that individuals with a Bachelor’s degree are more likely than their peers to use both the internet and traditional healthcare services as health resources. This study provides evidence in favor of the hypothesis that U.S. adults – and especially young adults with college degrees – are largely using the internet as a *complement* to the information and services provided by traditional medical providers, rather than a replacement.

## 1. Introduction

The internet has become a largely ubiquitous health information resource since the beginning of the 21st century, with 85 % of all American households reporting in-home internet access by 2018 (Martin, 2021). While detailed information about nearly all aspects of health and health conditions was historically almost exclusively the province of medical professionals, it is now accessible to anyone with internet access (Hardey, 1999). The quality of this information is not guaranteed, however, as the relatively unmoderated nature of the technology allows anyone with sufficient web or social media skill to contribute content. However, the wide spectrum of quality of online health information has not seemed to diminish enthusiasm for use of the internet as a health resource, with two-thirds of American adults now consulting the internet as their first source in their most recent search for health information (Finney Rutten et al., 2019). The expanding universe of online health information has drawn interest and concern from medical sociologists and practitioners alike.

Theoretical perspectives on the internet as a health information resource are conflicted between those who expect the internet to be a competitor to the traditional medical establishment (the ‘internet-as-threat’ view) and those who expect the internet to exist alongside, but not in competition with, traditional medical services (the ‘internet-as-complement’ view). Additionally, existing theories on fundamental causes of health disparities suggest that use of the internet as a novel health resource and the related effects on health behaviors may be concentrated among those with greater socioeconomic resources, despite the widespread proliferation of the technology (the ‘internet-as-resource’ view).

Despite the theoretical importance and potentially wide-reaching population health impacts, research attempting to quantify the population-level effects of internet use on health services use has been comparatively limited and whether these effects exist remains an open question (Conrad and Stults, 2010, p. 187). Suziedelyte’s (2012) investigation of the effects of internet use on count of general healthcare visits is a notable exception; however, the study period only covers the early years of the internet (2003–07) – before the widespread use of social media platforms like Facebook and Twitter to share health information and content of varying quality (Wang et al., 2019; Nan et al., 2021) – and uses only a binary measure of internet use. In contrast to Suziedelyte (2012), I do not use a causal approach, as the relevant theoretical perspectives do not specify a causal order but rather a set of plausible relationships between internet use and interaction with the medical establishment. For instance, individuals may use the internet before or after seeing a physician, but either use would support the internet-as-complement perspective since they use both sources of medical information in tandem, rather than one instead of the other.

In this study, I contribute to the understudied but critically important question of the relationships between the use of the internet as a health information resource and use of traditional health services. I first synthesize the existing theoretical perspectives on the internet as it may influence health behaviors and identify the hypothesized relationship each perspective yields. I then test each hypothesis using a series of regression analyses, which illustrate how different measures of internet use are associated with visits to health care professionals. Finally, I delve into the potential moderation of the relationship by age group or socioeconomic status using models featuring interaction effects. These findings contribute important empirical evidence to the prevailing debates about the role and dominance of the traditional medical establishment in the era of the internet.

## 2. Background

Over the past two decades, individuals have gained unprecedented access to a wealth of online health information of highly varying quality and with uncertain consequences for users’ health behaviors.

The sheer breadth of health information available to users online is tempered by the inconsistent quality of content in terms of scientific backing, with increasing concerns about the prevalence of low quality content (Suarez-Lledo and Alvarez-Galvez, 2021; Wang et al., 2019). Online health content can come from any individual or organization, ranging from accounts of patients’ and physicians’ personal experiences to postings from scientific institutions to commercial ventures, all of whom may lay claim to different forms of expertise around a particular health condition or topic. A study of online content containing advice about diabetes management and diagnosis found that personal recommendations and information about treatments or ‘cures’ could be “difficult to distinguish” from the health information promoted by official health authorities (Beguerrise-Díaz et al., 2017). The dispersed nature of online health information is seen by some as a strength of the internet as a health information resource, however. While individuals generally seek information online about singular health conditions of personal interest (Lemire et al., 2008; Miller et al., 2020), they value the greater availability of information online compared to other sources (Fox and Rainie, 2000), likely resulting in interactions with disparate and potentially conflicting information.

A burgeoning literature has examined the rise of the ‘lay expert’ in the digital age, wherein individual patients construct themselves as experts on their own health and health conditions, often based in extensive consumption of online health information and content (Prior, 2003; Naghieh and Parvizi, 2016). The interactions between healthcare professionals and these individuals are more likely to blur the traditional physician-patient power dynamic (Timmermans, 2020; Shachar, 2022; Wang et al., 2018), highlighting one pathway by which the internet as a health information resource may have changed the *experience* of health care visits. However, the relationship between the internet as a health information resource and *use* of formal health care services (as opposed to self-treatment or use of non-traditional means of treatment) is yet understudied and the current state of evidence is mixed. A study using longitudinal cohort data found that adults who were consistent users of the internet reported more positive health behaviors than non-users, including undergoing colorectal cancer screenings and other cancer-preventive health behaviors (Xavier et al., 2013). In contrast, a study using a spatial microsimulation approach found that internet use was associated with lower use of health services (Deetjen and Powell, 2016). Young adults themselves expressed how social media could provide both positive and negative influences on offline health behaviors, depending on the content (Vaterlaus et al., 2015) and the content creator (Durau et al., 2022). The mixed evidence on the relationship between internet use and health behaviors is likely due to counter-balancing trends, wherein certain types of use promote prosocial health behaviors and other forms of use promote negative health behaviors (Deetjen, 2016; Nettleton et al., 2005; Ziebland and Wyke, 2012). This study contributes to the literature by elucidating the relationship between internet use and the specific prosocial behavior of visits to healthcare professionals.

While the varying quality and breadth of online health information may limit its overall utility as a positive health resource, the increasing encroachment of the internet into terrain previously occupied only by medical experts has stoked the fire of ongoing debates about the offline influence of the internet on health behaviors and use of health services provided by traditional medical institutions (i.e., trained physicians and healthcare professionals operating in hospitals, clinics, or private offices).

### 2.1. Theoretical expectations for the relationship between internet use and health services use

Three general theoretical perspectives are key to framing and understanding the potential relationship between the internet as a health information resource and offline health service use behaviors. First, the internet-as-threat perspective argues that open access to information once largely exclusive to medical professionals will empower individuals to manage their own health and treatment, lessening their use of health services. Second, the internet-as-complement perspective generally expects that, while patient-physician interactions may change in favor of more empowered patients, patterns of overall health services use and the predominance of the traditional medical establishment will persist and adapt amidst the proliferation of the new technologies. Finally, the internet-as-resource perspective draws on fundamental cause theory and posits that the adoption of the internet as a potentially health-promoting technology will likely be stratified such that more well-resourced individuals are earlier or more responsive adapters and thus the relationship between internet use and health services use may be pronounced or concentrated among this group.

#### (1) Internet-as-threat

The relatively open, unmoderated nature of the internet has been theorized to be a threat to the medical establishment since the advent of the technology (Hardey, 1999; Cotten, 2001), as individuals may increasingly rely on information from ‘illegitimate’ sources instead of consulting physicians and other medical professionals. This threat is made manifest in the notion of the ‘lay expert,’ or individuals who have “extensive knowledge of their own lives and the conditions in which they live” and “can … turn themselves into experts in order to challenge medical hegemony” (Prior, 2003, p. 45). A growing body of evidence highlights the emergence of lay expert communities, facilitated by the internet (Naghieh and Parvizi, 2016), with participants creating and disseminating information and knowledge based on their personal experiences of a health condition (e.g., Campbell, 2021; Au and Eyal, 2022; Bellander and Landqvist, 2020). Unfettered access to this health information and co-constructed knowledge as well as the ‘lay expert’ perspective may lead individuals to feel more empowered to define and manage their own health states or conditions and to be less likely to seek out traditional medical services. Given the costs and barriers to access associated with high-quality health services – particularly for racial minorities (Fiscella and Sanders, 2016; Mahajan et al., 2021), rural residents (Douthit et al., 2015), and uninsured adults (Choi et al., 2020; Liang et al., 2019) – the wealth of information provided by the internet may appear to be a desirable and feasible alternative to traditional healthcare.

Some studies have found empirical evidence in favor of this theoretical perspective: Lowrey and Anderson (2006) found “increased access and use of online health information is related to the belief that doctors may be bypassed in the pursuit of medical information and diagnoses.” One Pew Research Center Study found that in 2013, more than one-third of American adults who diagnosed themselves with a condition based on online information say they did not visit a clinician to get a professional opinion (Fox and Duggan, 2013). A study of French young adults found that approximately 30 % reported using the internet for health purposes instead of seeing a doctor (Beck et al., 2014).

##### The internet-as-threat perspective suggests Hypothesis 1:

Use of the internet as a health information resource will be associated with reduced use of health services (e.g., lower probability of visiting a healthcare professional and lower count of visits).

#### (2) Internet-as-complement

Other scholars are skeptical that lay expertise, particularly of the internet variety, can truly infringe upon the predominance of the traditional medical establishment. First, while the internet is a repository of health information, it can also facilitate finding local health care providers or specialists. Second, the cultural authority afforded to medicine and medical practitioners appears to prevail even in the online space, as users place a high level of trust and importance on online information contributed by physicians or health professionals (Lemire et al., 2008) and even adopt medical perspectives and language in online patient communities (Pitts, 2004; Bellander and Landqvist, 2020). Third, health care professionals in the internet age may adapt to maintain their own relevance (Forgie et al., 2021). Blumenthal (2002) predicts that, while there will likely be a decline in the knowledge asymmetry between patient and physician, physicians will “continue to offer genuine technical competence that will be difficult, or impossible, to replicate from other sources” and may come to adopt a role similar to consultant (p. 543). While healthcare professionals might give up some ground on their authority over health information, they will likely retain authority over physical services (e.g., blood tests, prescriptions) for the foreseeable future (Lee, 2008). In summary of this perspective, Epstein and Timmermans (2021) conclude that while “the online revolution appears to threaten the cultural authority of medicine,” the internet is likely to be a complement to traditional medical expertise rather than a replacement (see also DiMaggio et al. (2001) and Stevenson et al. (2021)). Suziedelyte’s (2012) empirical study of the early internet (2003–2007) supports this perspective with the finding that online health information-seekers have higher demands for health care; however, the rapid development of the internet in the two decades since the study’s data collection raises concerns about the continued validity of the findings.

##### The internet-as-complement perspective suggests Hypothesis 2a:

Use of the internet as a health information resource will be associated with greater use of health services (e.g., higher probability of visiting a healthcare professional and greater count of visits).

#### (3) Internet-as-resource

**Fig. 1.**
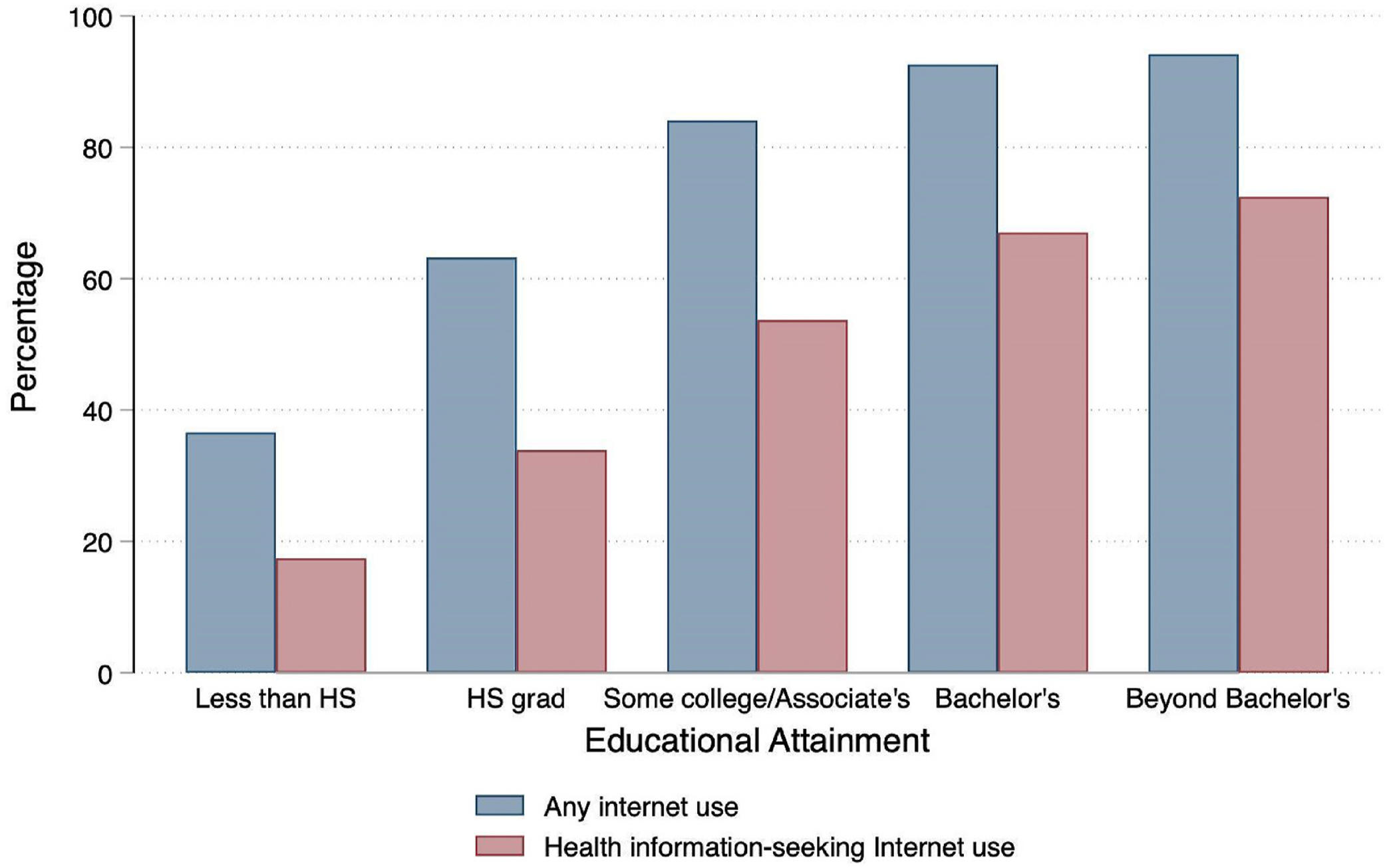
Rates of general and health information-seeking internet use by educational attainment, pooled NHIS 2012-2018.

The logic of Fundamental Cause Theory argues that well-resourced individuals – for instance, those with more education, wealth, or power – tend to have better health than their disadvantaged counterparts (Link and Phelan, 1995; Phelan et al., 2010). Moreover, this socioeconomic gradient in health outcomes persists and reappears because these fundamental resources can be used flexibly to gain health advantages, even in novel situations. Highly educated individuals are more likely to be early adopters of potentially beneficial health technologies (Glied and Lleras-Muney, 2008; Tehranifar et al., 2009) and protective health behaviors in response to new information, as in the case with tobacco use over the course of the late 20th century (Cummings and Proctor, 2014). Cotten and Gupta (2004) find some evidence to support this theory, with bivariate analyses indicating lower levels of education or income were associated with a lower likelihood of internet utilization in general and to seek out health information. Consequently, well-resourced adults may be both more exposed to and responsive to online health information that seems relevant to their personal health (Fig. 1). Moreover, they may be more inclined to accumulate and consolidate resources, thus opting to use *both* the internet and healthcare professionals as sources of health information. Empirical evidence strongly supports an education gradient in preventive services uptake (Sabates and Feinstein, 2006; Lorant et al., 2002; Avitabile et al., 2008). Fundamental Cause Theory would thus predict that, while well-resourced adults are less likely than their peers to become ill, they are also more likely to use all the health resources at their disposal to maintain that advantage. This would result in a stronger positive association between health information-seeking internet use and physician visits for higher-SES groups, particularly as the outcome excludes emergency visits.

##### The internet-as-resource perspective suggests Hypothesis 2b:

Use of the internet as a health information resource will be associated with greater use of health services, but the relationship will be stronger among individuals with greater socioeconomic resources (i.e., there will be a significant positive interaction effect between education and health information-seeking internet use).

### 2.2. The importance of age

**Fig. 2.**
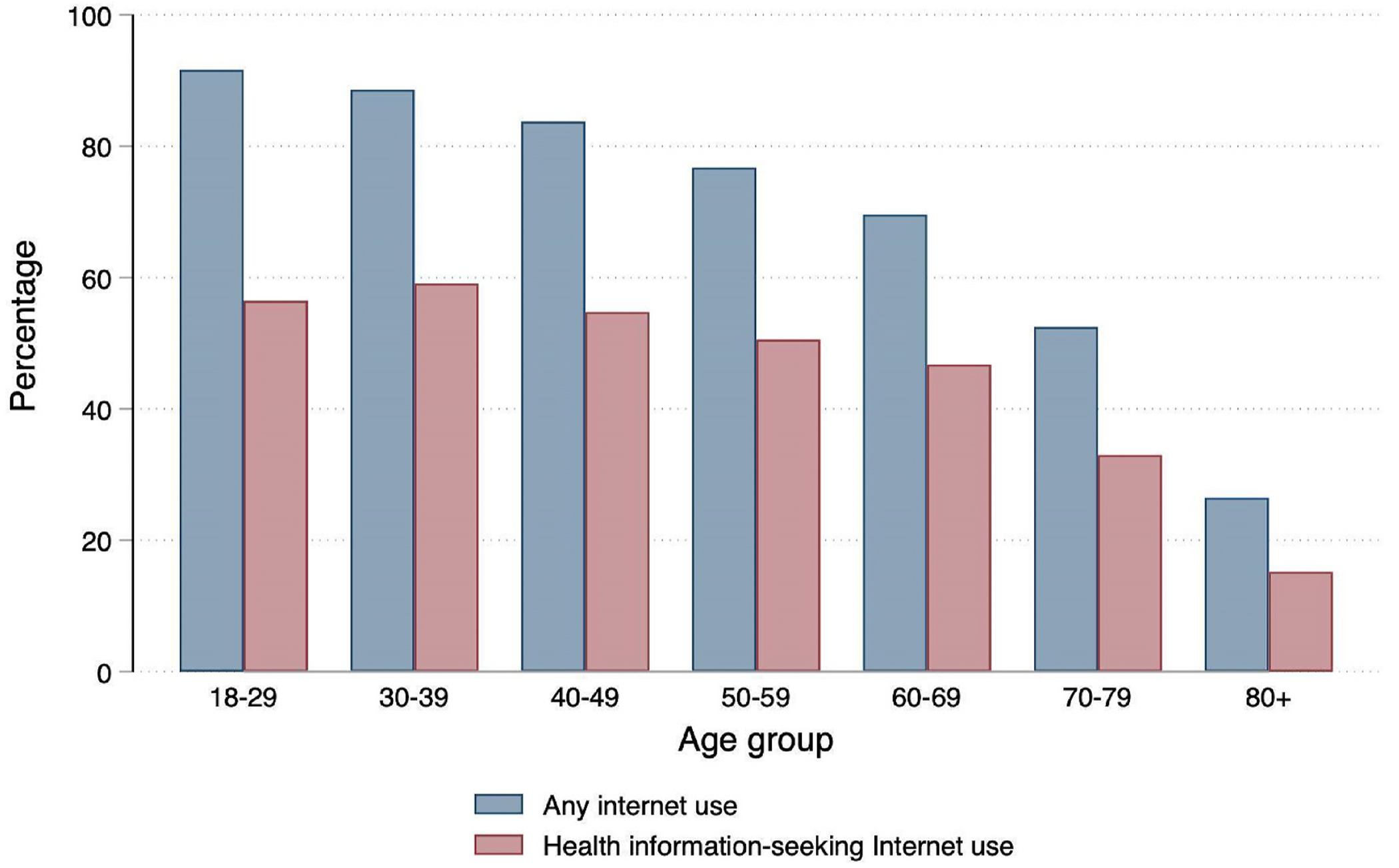
Rates of general and health information-seeking internet use by age group, pooled NHIS 2012-2018.

Compared to those for whom it debuted during later life stages, individuals who ‘grew up’ with the internet –who are currently young to early middle-aged adults – show both higher rates of internet use and differentiated patterns of use in which online platforms they frequent (Gottfried, 2024). Young adults are generally more likely to use the internet at all and, in particular, to seek out health information (Rice, 2006) (Fig. 2). One study found the mean age of adults who used the internet to seek health information was eleven years younger than those who used other sources (Cotten and Gupta, 2004). In a nationally representative study of older adults, Zulman et al. (2011) found only 16 % of their sample reported having “a lot” of trust in the internet as a health resource, with 37 % saying “somewhat” and 47 % having limited or no trust. Interaction effects with age have previously been found in the internet-health relationship – for young adults, ‘active’ social media use is associated with better mental health outcomes while ‘passive’ social media use is associated with worse outcomes; for older adults, this trend is reversed (Lewin et al., 2022). These trends suggest that younger adults may be more responsive, for better or for worse, to online health information than older adults.

#### The current body of research into age patterns of internet use suggests Hypothesis 3:

There will be a significant interaction effect of internet use with age such that any relationship between health information-seeking internet use and physician visits will be strongest among younger adults.

## 3. Data and methods

### 3.1. Data

I use data from the pooled 2012–2018 waves of the National Health Interview Survey (NHIS), a nationwide survey of non-institutionalized U.S. civilians conducted by the National Center for Health Statistics (Blewett et al., 2023). The NHIS collects data on a number of aspects of population health, including individuals’ medical history, healthcare services utilization, and health-related behaviors. These years were chosen as they were the most recent surveys to contain all health and internet use measures of interest. To construct the main analytic sample, I included respondents from each survey year (2012–2018) who were aged 18 years and older who reported key sociodemographic information (race, ethnicity, sex, age, marital status, education, and region) and self-rated health. I used multiple imputation through chained equations to create ten imputed data sets with complete data for the other variables in the model, which I used for model estimation (n = 500,225). For the models using the focal predictors of frequency of internet use, I subset only to respondents who reported any use of the internet to seek out health information. Minimal differences were found in the characteristics of the full and analytic samples. Estimation of the same models on a sample only of respondents with complete information for all variables provided the same pattern of results. The results are consistent when the model is run for each individual survey year; pooling was used to increase statistical power. Sampling weights to account for survey design and nonresponse were used in all models.

### 3.2. Measures

The primary outcome of this study is derived from respondents’ self-reported total count of visits in the past year to see a physician or other healthcare professional about their own health. This count does not include emergency visits (i.e., overnight hospitalizations or visits to hospital emergency rooms), dental visits, home visits, or telephone calls. In the main analyses, this variable is dichotomized into two options: no visits and one or more visits. In a supplementary analysis, this variable is treated as quasi-continuous, with the options of no visits, 1 visit, 2–3 visits, 4–5 visits, 6–7 visits, 8–9 visits, and 10+ visits.

I use three measures of internet use to more fully capture the degree of engagement with online health information and resources. The main analysis uses a dichotomous measure of whether or not a respondent used the internet to look up health information in the past 12 months (yes = 1). Next, I subset the model to only include respondents who reported any use of the internet to search for health information and use a measure of frequency of internet use (daily, weekly, monthly, yearly) as the focal predictor. Finally, I further subset to only daily internet users and use a count of uses of the internet per day as the focal predictor.

I control for a series of sociodemographic variables as potential confounders, as they have associations with both internet access and use (Estacio et al., 2019) and health services access (Ferrer, 2007; Lasser et al., 2006). Sociodemographic variables included in the main models were age, sex, race, ethnicity, marital status, census region of residence, health insurance, family income, and education. Age was a continuous variable, with respondents’ age range between 18 and 85 years old (top-coded). Sex was dichotomized as male or female (male = 1). Respondents self-reported their racial identity as “White only,” “Black/African American only,” “American Indian/Alaska Native only,” “Asian only”, or “Multiple race.” Ethnicity was a dichotomous variable indicating if the respondent considers themselves to be Hispanic or Latino (yes = 1). Marital status was categorized into “married,” “divorced or separated,” “widowed,” or “never married.” Census regions of residence included northeast, north central, south, and west. Respondents’ health insurance status was categorized as no insurance, private insurance only, public insurance only, and both private and public insurance. Family income was categorized into less than $50k, $50-99k, or at least $100k, adjusted for inflation. Respondents’ education was categorized as less than high school attainment, high school graduate, some college or associate’s degree, Bachelor’s degree, or attainment beyond Bachelor’s degree. Additionally, I control for survey year (2012–2018) in all models.

To control for need for (non-emergency) medical care, I included a number of variables measuring health conditions and behaviors. Health condition variables included self-rated health (“excellent,” “very good,” “good,” “fair,” or “poor”), body mass index (normal, underweight, overweight, or obese), and chronic condition status, constructed in line with Boersma et al. (2020) as categorical with respondents reporting 0, 1, or at least 2 chronic conditions from a list of ten (hypertension, coronary heart disease, stroke, diabetes, cancer, arthritis, hepatitis, weak or failing kidneys, adult asthma, or COPD). Health behavior variables included smoking behavior (“never smoked,” “current smoker,” “former smoker,” or “unknown”) and alcohol consumption (number of days per week during the past year that the respondent drank alcoholic beverages, ranging from 0 to 7; abstainers were coded as 0).

Informed by Andersen (1995), to account for individuals’ access to health services, I included a dichotomous measure of if a respondent reported at least one place that they usually go to when sick or in need of advice about their health (such as a clinic, health center, doctor’s office or HMO, hospital emergency room, or hospital outpatient department) (yes = 1).

In supplementary analyses, I include two dichotomous measures of individuals’ use of telemedicine: use of the internet to make an appointment with a healthcare professional (yes = 1) or to fill a prescription (yes = 1).

### 3.3. Analytic strategy

I first provide weighted descriptive statistics on all model variables (Table 1). I then conduct a series of logistic regressions to assess the relationship between internet use to seek health information and use of traditional medical services, operationalized as non-emergency visits to a doctor or other healthcare professional in the past year (Table 2). I further investigate the focal relationship by conducting a series of regression models using more granular measures of internet use among users (Table 3). I sequentially include covariates associated either with access to the internet or need for and access to health services.

In Model 1, I include sociodemographic characteristics (age, race, sex, ethnicity, region of residence, marital status, education, family income, health insurance status/type). In Model 2, I add self-rated health, count of chronic conditions, body mass index (BMI) category, smoking behavior, and alcohol consumption. In Model 3 (fully specified), I add a measure of reporting a usual place of medical care. Additionally, I rerun Model 3, subset to only individuals reporting any use of the internet as a health information resource and using more granular measures of internet use frequency: first, a measure of frequency of internet use (daily, weekly, monthly, yearly) as the focal predictor; second, a count of uses of the internet per day among daily users as the focal predictor.

In Model 4, I test for interactions between age group and health information-seeking internet use. In Model 5, I test for interactions between educational attainment and health information-seeking internet use. In line with the current best practices for interpreting interaction effects in non-linear models, I estimate and evaluate first and second differences rather than relying on the significance of the product term coefficient (Mize, 2019; Mustillo et al., 2018; Ai and Norton, 2003).

I conduct three main supplementary analyses in the interest of testing the sensitivity of the main model specifications: (1) running Model 3 as a linear regression using the quasi-continuous measure of count of healthcare visits as the outcome; (2) running Model 3 including two telemedicine variables described above; (3) running Model 5 substituting educational attainment for family income. Results of all sensitivity tests are included in the Appendix. All analyses were conducted in Stata Version 18.0.

## 4. Results

Table 1 shows descriptive characteristics of the full sample and analytic sample.

In the full sample, the majority of adults (83 %) reported at least one visit to a doctor or other healthcare professional in the past year. Unsurprisingly, there was a roughly linear increase with age in the proportion of adults with any visit, ranging from 73.8 % of 18–29 year olds to more than 94 % of adults aged 80 and older. Among those reporting any healthcare visits, 21.1 % reported one visit, 32.1 % reported 2–3 visits, 17.2 % reported 4–5 visits, and 29.6 % reported six or more visits. Individuals reporting their health to be fair or poor had both a higher proportion reporting any visit and a greater number of visits than individuals with better self-rated health.

Over three-quarters (75.8 %) of adults reported any internet use, ranging from 91.6 % of 18–28 year olds to just over half (52.5 %) of adults aged 70–79 and just over a quarter (26.5 %) of adults aged 80 and older. About half (49.1 %) of adults reported using the internet in the past year specifically to look up health information; the age gradient was less steep in this measure, with about half of adults in all age groups under the age of 70 reporting use of the internet for this purpose. The rates of health information-seeking internet use were distinctly stratified by education, with 17.4 % of adults with less than high school education, 33.9 % of high school graduates, 53.7 % of adults with some college or an associate’s degree, 70 % of college graduates, and 72.4 % of adults with education beyond a Bachelor’s degree reporting use of the internet as a health information resource.

Adults who reported their health as excellent or very good had the highest rates of use of the internet to seek health information, at 53.8 % and 54.7 % respectively, compared to less than 40 % of adults who reported their health as fair or poor.

Table 2 shows results from Models 1–4 using the analytic sample.

Use of the internet to find health information was consistently significantly associated with increased odds of reporting a visit to a doctor or other healthcare professional across Models 1–3. Respondents who reported any use of the internet to find health information in the past year reported higher odds of any healthcare visits than those who didn’t: odds were 78.4 % higher in model 1 and slightly attenuated to 67 % higher in model 3 after health and healthcare access variables were included (p < .001 for all three models). These findings provide support for Hypothesis 2a and the internet-as-complement perspective, in contrast to Hypothesis 1 and the internet-as-threat perspective.

When Model 3 was run among users of the internet as a health information resource (Table 3), daily internet users (reference group) have significantly higher odds of any healthcare visits than weekly users (OR = 0.791, p < .001) and higher odds than monthly or yearly users (odds ratios of 0.884 and 0.775, respectively; p > .05). Among daily users, each additional instance of internet use per day was associated with a 0.2 % increase in the odds of any healthcare visit, though this finding was not statistically significant.

**Fig. 3.**
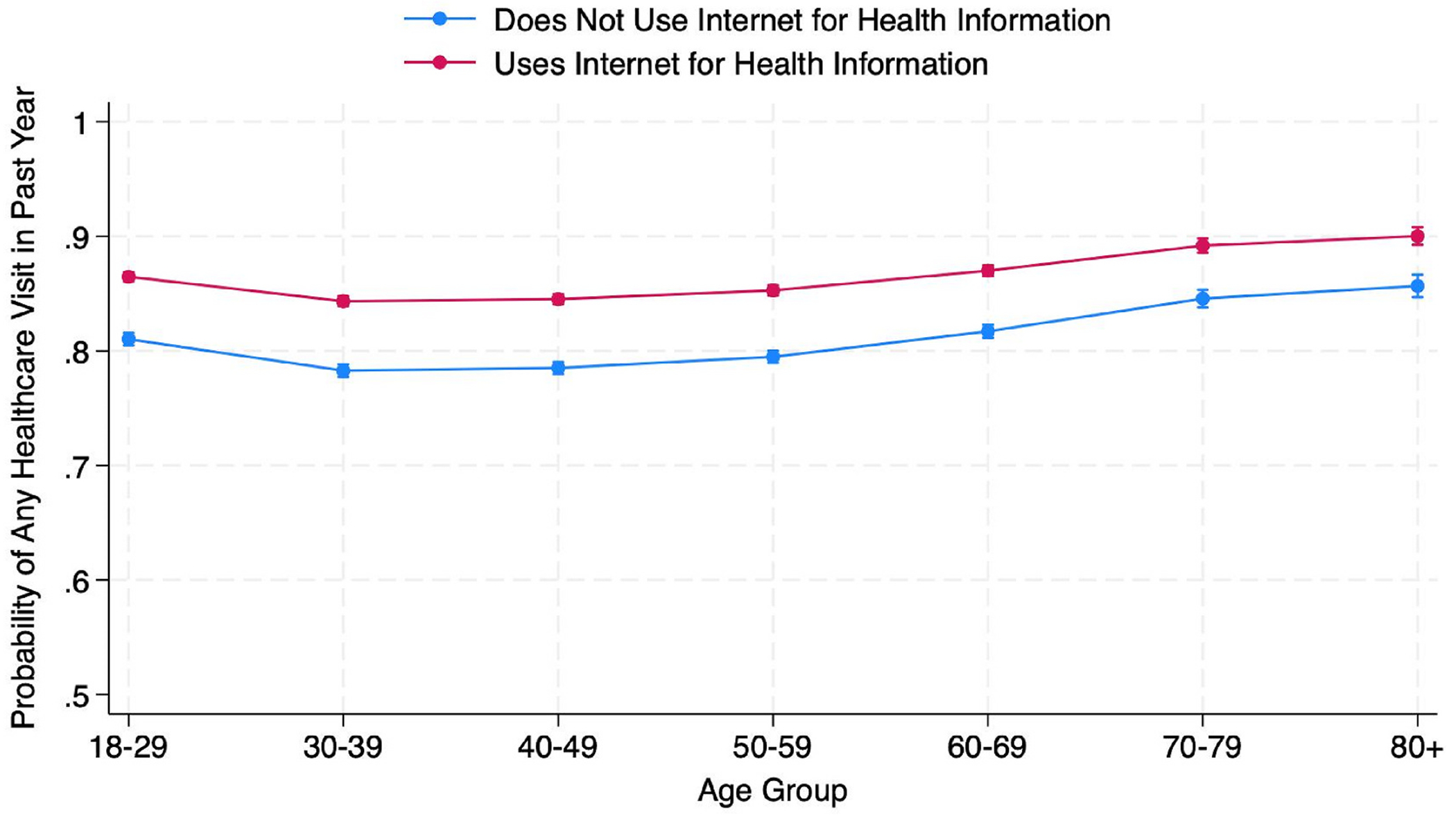
Predicted probabilities of at least one physician visit in the past year by age group and use of internet for health information.

**Fig. 4.**
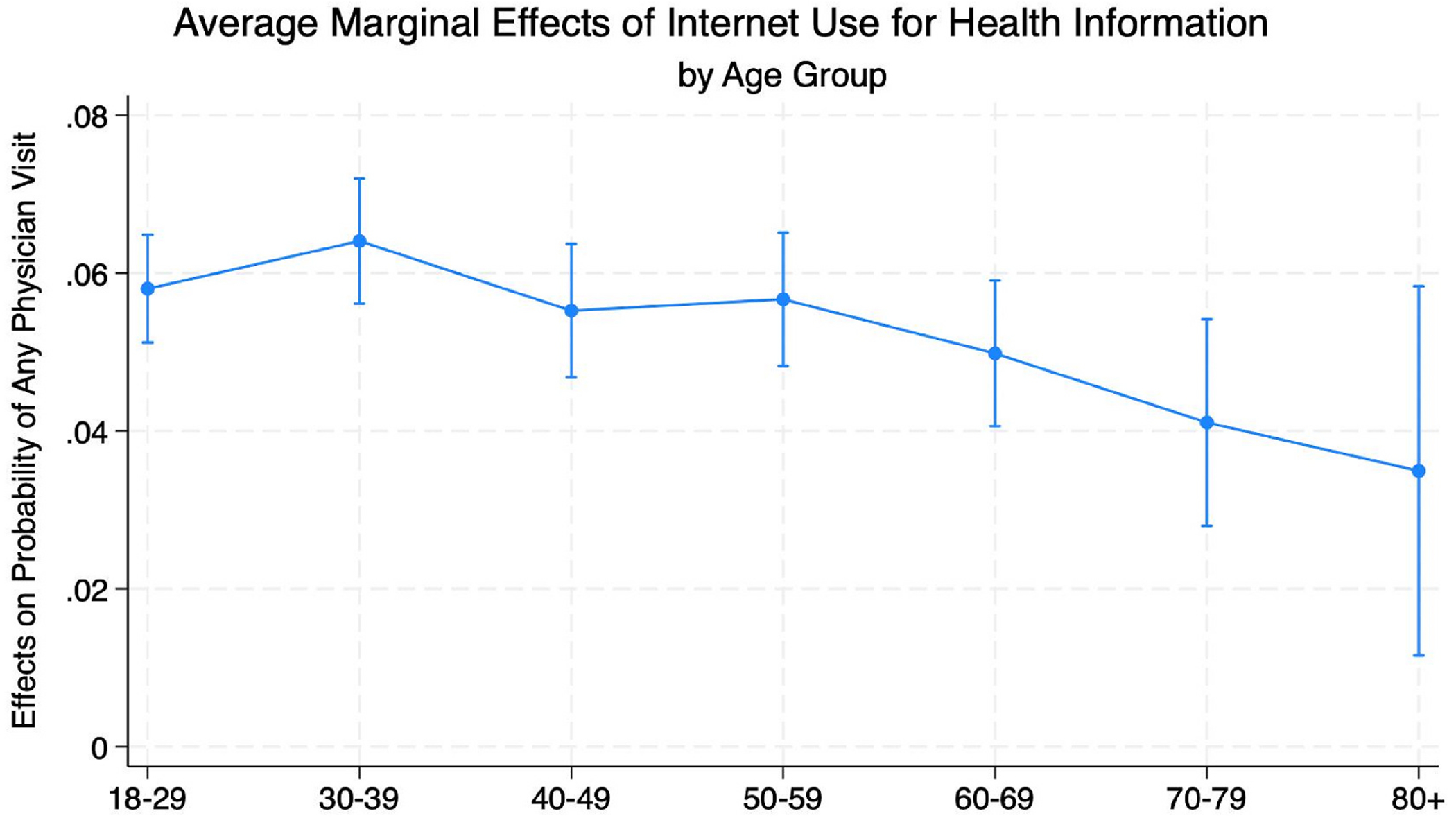
Average marginal effects of use of the internet for health information on probability of physician visit, stratified by age group.

In Model 4, there was some evidence of an interaction between age group and health information-seeking internet use in its effect on probability of visiting a physician at all (Hypothesis 3) (Fig. 3) (Appendix Table A1). The first differences were all significant, indicating that within each age group, respondents who used the internet to seek health information had significantly greater probabilities of visiting a physician at all (p < .05). There were four sets of significant differences (p < .05): between age groups 18–29 and 70–79 and between age group 30–39 and all older adult groups (60–69, 70–79, and 80+), such that the average marginal effects of internet use to seek health information on probability of physician visits was higher among the younger adults than the older adults (Fig. 4).

**Fig. 5.**
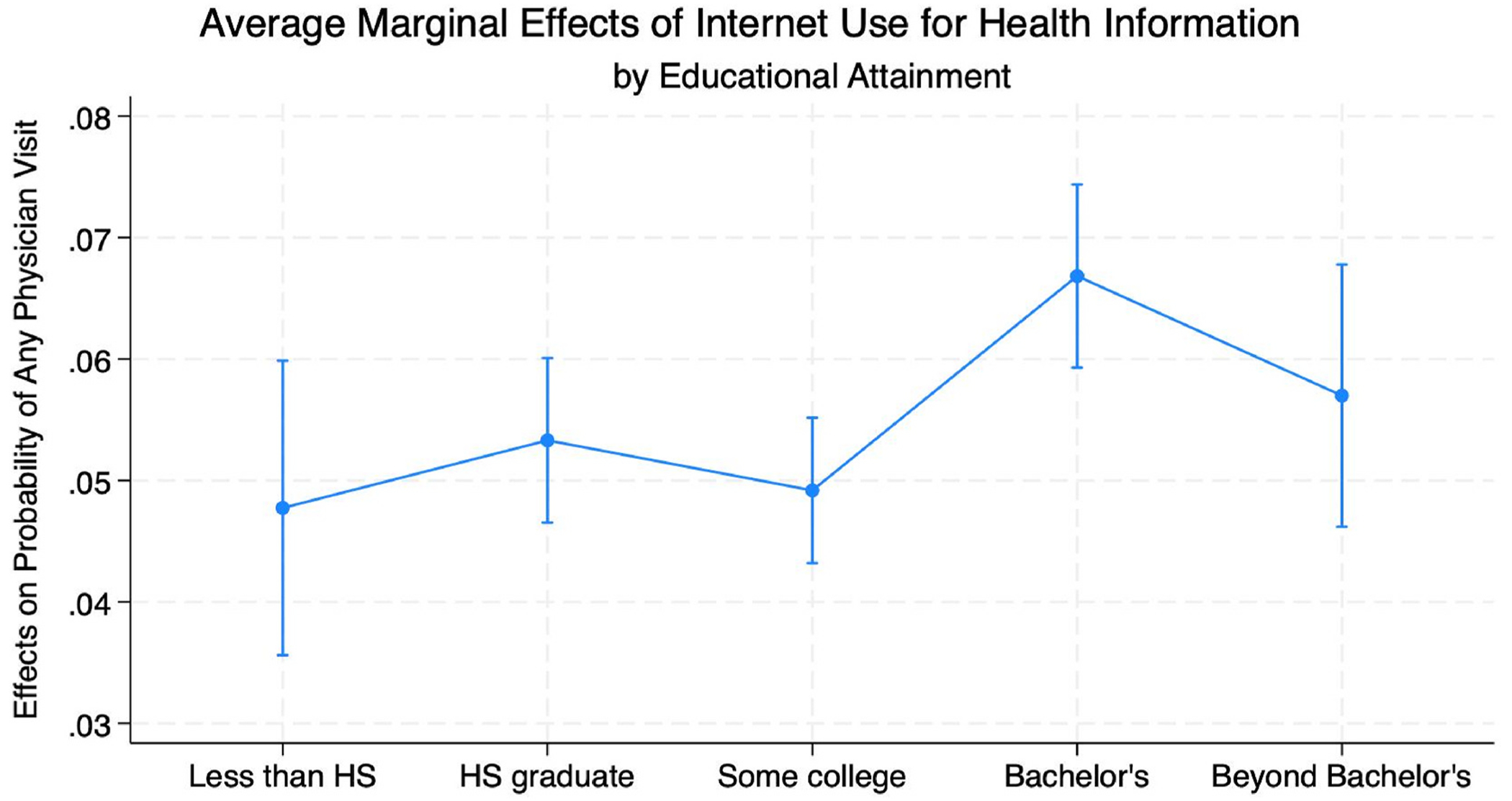
Average marginal effects of use of the internet for health information on probability of physician visit, stratified by educational attainment.

In Model 5, there was some evidence to support an interaction between education status and health information-seeking internet use, with the strongest relationship among respondents with a Bachelor’s degree (Hypothesis 2b) (Appendix Table A2). The first differences (e.g., difference between predicted probabilities by health information-seeking internet use status within education group) were statistically significant (p < .05) for all groups, further adding evidence in support of the internet-as-complement perspective (Hypothesis 2a). Compared to respondents with less than high school, a high school degree, or some college, respondents with a Bachelor’s degree had significantly larger marginal effects of health information-seeking internet use on probability of any healthcare visits (test of second differences, p < .05) (Fig. 5).

### 4.1. Sensitivity tests

**Fig. A1.**
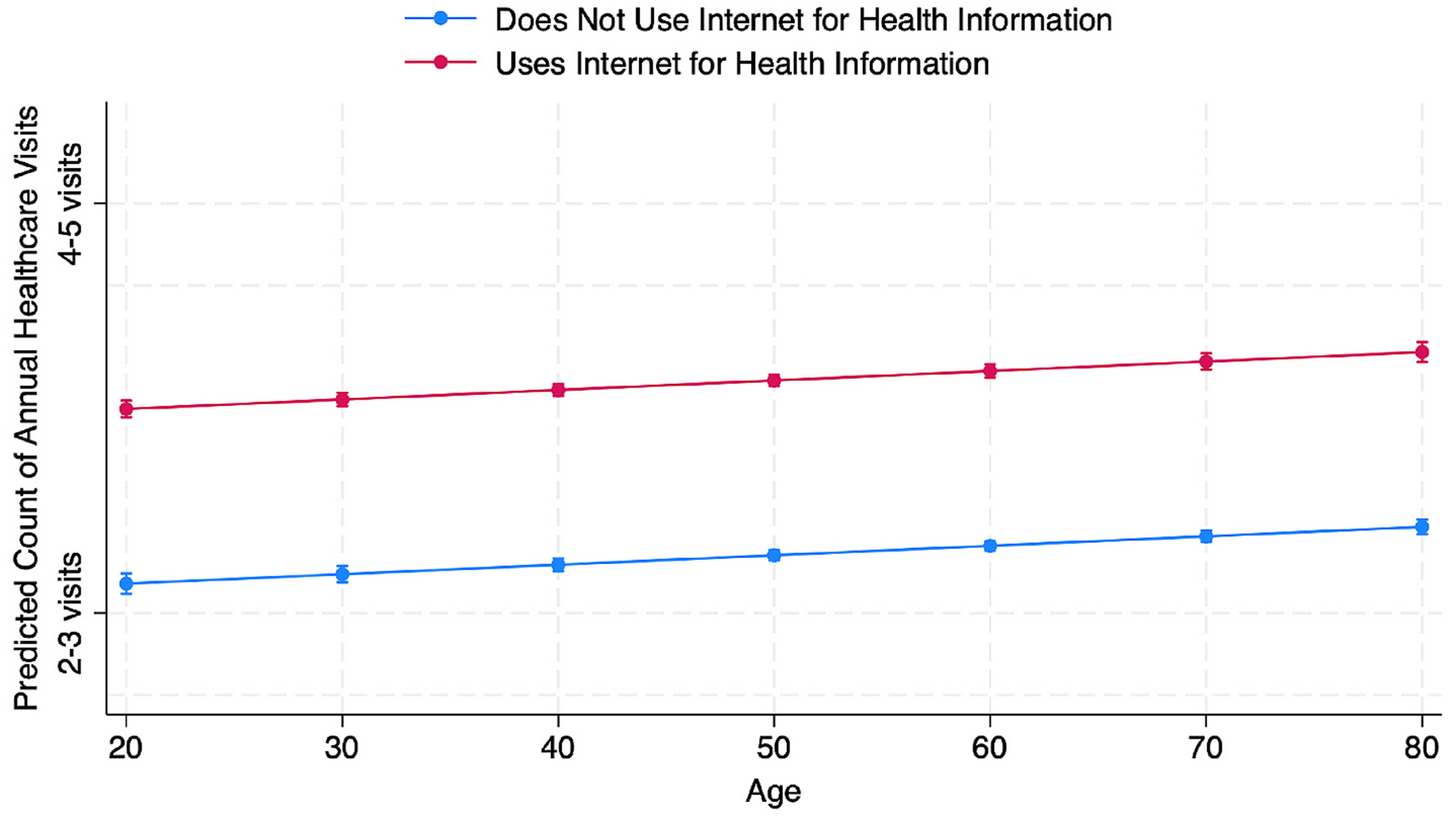
Predicted count of at least healthcare visit in the past year by health information-seeking Internet use status and age group.

When Model 3 was repeated using a linear regression and the outcome was operationalized as count of visits to a doctor’s office or healthcare professional, respondents who used the internet to find health information reported, on average, a 0.43 unit increase in the measure of visit counts, controlling for any internet use (p < .001) (Appendix Table A3). Among all age groups, this translates to a change in predicted count of healthcare visits from 2 to 3 visits to closer to 4–5 visits per year (Appendix Figure A1).

When Model 3 was rerun including the two telemedicine measures, the relationship between health information-seeking internet use and visits to healthcare professionals was maintained (OR = 1.502, p < .001) (see Appendix Table A3).

**Fig. A2.**
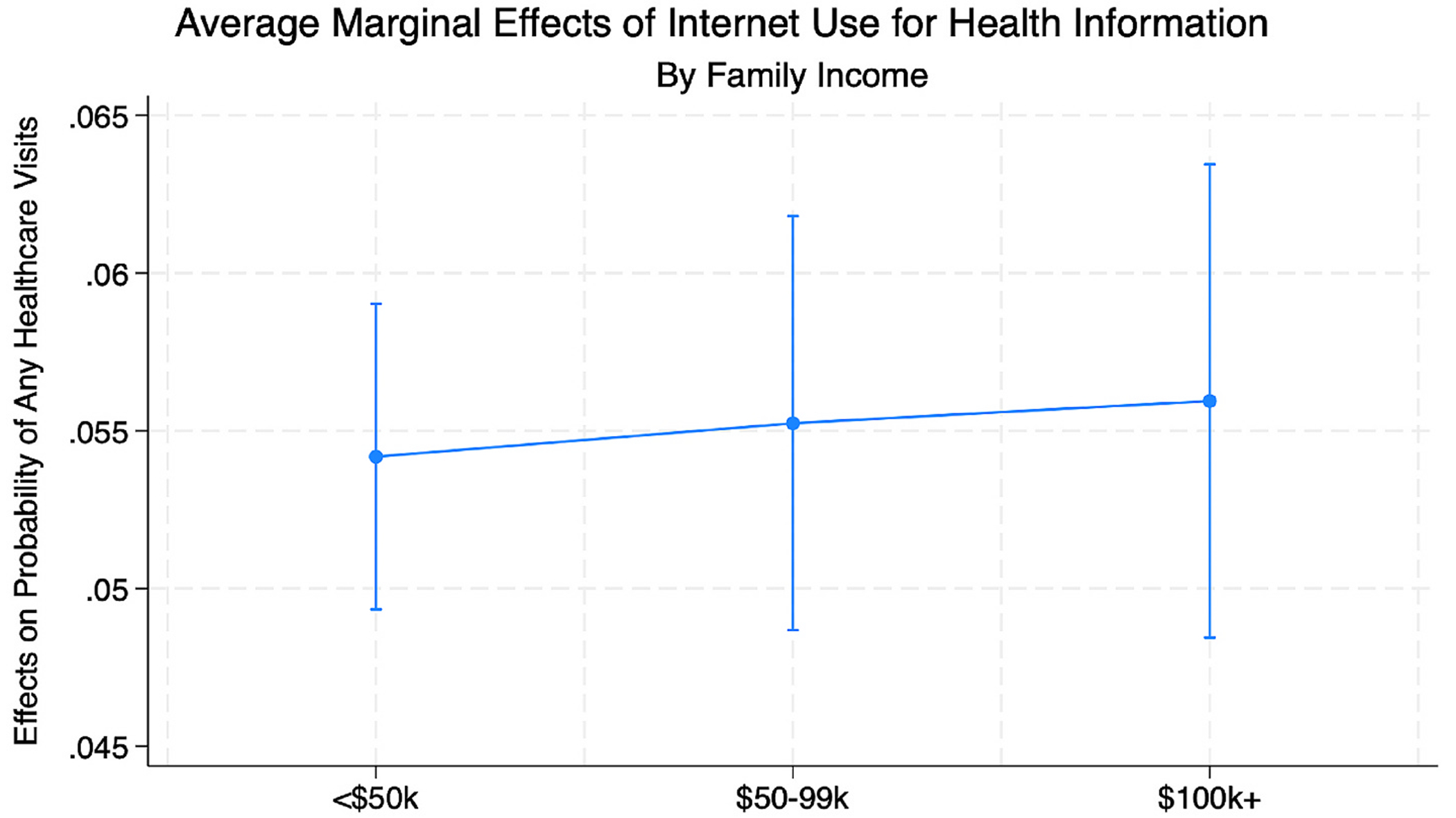
Average marginal effects of health information-seeking Internet use by family income status.

When Model 5 was run featuring an interaction between income and health information-seeking internet use rather than education, first differences were statistically significant for all groups but second differences were not, indicating that while the relationship between use of the internet as a health resource and use of healthcare services may differ in magnitude by educational status, it does not differ by income status (see Appendix Figure A2.)

## 5. Discussion

The internet provides individuals with access to a nearly infinite amount of content on medical and health issues. How, when, and to what extent the use of the internet both generally and specifically to seek health information affects offline behaviors and well-being is an open question of deep importance to population health. In this study, I find that use of the internet to seek health information is associated with significantly higher odds of visiting a doctor or healthcare professional at all and higher counts of visits. This relationship is strongest among younger adults, adults with a Bachelor’s degree, and daily internet users.

These findings align with the ‘internet-as-complement’ theoretical perspective of the internet’s influence on the social world, which posits that internet use tends to complement, rather than replace, traditional forms of medical expertise (Timmermans, 2020). Indeed, while the amount of available information is staggering, individuals still retain a degree of agency on the internet in what they look for, what content they interact with or ignore, and ultimately in limiting the information to which they are exposed (Schroeder, 2018). Thus, while a compelling theoretical perspective suggests that the internet as a source of health information content has changed public perceptions of the medical establishment and potentially patients’ interactions with medical professionals (e.g., Forgie et al., 2021; Deetjen, 2016; Broom, 2005), the above analyses illustrate that at the population level, the internet has not replaced and instead appears to supplement use of traditional medical services. A burgeoning literature on ‘lay experts’ using the internet to build their health knowledge highlights how patient-physician interactions and power dynamics are changing in the age of the internet (Broom, 2005; Radin, 2006; Bellander and Landqvist, 2020). While this literature highlights that the internet as a health resource can change *how* individuals interact with their healthcare providers, the central finding of this study suggests that the internet as a health resource still facilitates *use* of healthcare providers – essentially, the experience within the doctor’s office may have changed for internet users, but they are still going there in the first place.

Since content on the internet is only lightly moderated, if at all, the health information or perspectives from competing sources may be difficult to interpret or form definitive conclusions about one’s own situation (Cotten, 2001); in the face of this uncertainty and discord, individuals may be more inclined to rely on more ‘traditional’ sources of medical authority who can consider the complexities of their unique case. Healthcare professionals also retain the authority to prescribe certain medications, which often necessitate an appointment and formal diagnosis regardless of an individual’s own knowledge of their condition. The increasing availability of telemedicine options – from online scheduling of in-person visits to virtual appointments with healthcare professionals – could also contribute to the internet as a ‘one stop shop,’ wherein individuals seeking health information are exposed to or motivated to use health services, based either online or offline (Blumenthal, 2002). However, the persistence of this positive correlation in models that control for telehealth behaviors suggests that use of the internet as a *medium* to access health services is not driving the central finding, which assesses use of the internet as a health information *resource* (see Appendix Table A3).

In addition to the central finding of a positive relationship between internet use and health services use, I find that this relationship is strongest among individuals with a Bachelor’s degree; however, I found no significant interactions with income, in contrast to the expectations of Fundamental Cause Theory. One potential mechanism explaining these findings could be the link between greater educational attainment and greater health literacy (Friis et al., 2016; Clouston et al., 2017). ‘Health literacy’ as a concept generally refers to individuals’ capacity to seek out and utilize health information to make decisions about their health within the context of social and institutional environments (Lee et al., 2004; Samerski, 2019). In the context of an information-rich internet landscape, the greater health literacy associated with higher levels of education may facilitate comfort in navigating and synthesizing the many sources of health information online (Nielsen-Bohlman et al., 2004; Estacio et al., 2019). Further research into this potential operation of this mechanism is warranted. While these findings suggest that the internet is largely not used by *any* socioeconomic group as a replacement for traditional health services, it may exacerbate existing health inequalities as highly educated adults appear to be both more exposed to and more inclined to use online health information in conjunction with traditional health services.

This study must be considered within the context of limitations. First and foremost, the use of high-quality nationally representative survey data limits the degree of specificity in measuring health information-seeking internet use. The primary measure used in this study only captures if respondents use the internet to seek online health information but does not provide further information about what content is consumed by respondents. In supplementary analyses, I used a measure of internet use frequency and controlled for telehealth behaviors. The persistence of the significant focal relationship in these models adds further evidence that online health information seeking has a positive relationship with healthcare visits beyond the use of the internet as a medium for accessing those healthcare services. However, this still does not provide direct evidence about what kinds of online health content are driving the positive association with health services use. This limitation of existing data sources highlights the pressing need for future research and surveys designed to capture online experiences of health information and content and the ways in which these experiences interact with offline health behaviors. Considering this, the central findings of the study should be understood as the *average* relationship between use of the internet for health information and use of healthcare services among American adults. Finally, the survey years included do not cover the COVID-19 pandemic era and therefore the conclusions do not extend to COVID-specific information seeking or physician visits.

Future research could further delve into the mechanisms driving the relationship between use of the internet as a health information resource and healthcare services use. In particular, researchers could assess if exposure to online health information contributes to early detection of health conditions and ultimately better outcomes. The relationship between use of the internet to seek health information and propensity to seek health services may also differ by the kind of healthcare visit (e.g., general ‘well visits’ versus concerns about a specific condition or symptom). Additionally, future studies could assess potential cohort effects – that is, if current young adults’ patterns of health information-seeking internet use persist into older ages. Ultimately, the strength of the focal association in this paper is compelling evidence that health information on the internet is sought by the general population as a supplement to – rather than replacement of – healthcare professionals’ expertise and authority.

## Figures and Tables

**Table 1:** Descriptive statistics of key study variables in pooled, weighted 2012–2018 NHIS sample.

Variable	Mean/Proportion (SD in parentheses)
Doctor’s visit at all	82.9 %
Office visit count	2.44 (1.92)
–None	17.1 %
–1 visit	17.5 %
–2-3 visits	26.6 %
–4-5 visits	14.2 %
–6-7 visits	6.7 %
–8-9 visits	3.5 %
–10+ visits	14.3 %
Use internet at all	75.8 %
Use internet for health info	49.1 %
Sex (male)	48.2 %
Region	
–Northeast	18.0 %
–North central	22.1 %
–South	36.8 %
–West	23.0 %
Race	
–White	79.1 %
–Black	12.3 %
–AI/AN	1.0 %
–Asian	5.9 %
–Multiple	1.8 %
Hispanic	15.6 %
Age	47.08 (18.05)
–18-29	21.4 %
–30-39	16.9 %
–40-49	16.9 %
–50-59	17.8 %
–60-69	14.4 %
–70-79	8.1 %
–80+	4.6 %
Marital status	
–Married	54.1 %
–Divorced	12.7 %
–Widowed	6.0 %
–Never married	27.2 %
Health insurance	
–None	14.3 %
–Private only	72.2 %
–Public only	13.0 %
–Private and Public	0.5 %
Family income	
–<50k	42.4 %
–50-99k	30.7 %
–100k+	26.9 %
Education	
–Less than HS	12.8 %
–HS grad	26.4 %
–Some college/associate’s	30.3 %
–Bachelor’s	19.4 %
–Beyond Bachelor’s	11.0 %
Self-rated health	2.26 (1.06)
1/excellent	28.7 %
2/very good	32.1 %
3/good	26.7 %
4/fair	9.7 %
5/poor	2.9 %
Chronic condition category	
–0	52.1 %
–1	26.6 %
–2+	21.3 %
Alcohol days per week	0.978 (1.80)
Smoker	
–Never	59.8 %
–Current	16.4 %
–Former	23.4 %
–Unknown	0.4 %
BMI	
–Normal	34.2 %
–Underweight	1.8 %
–Overweight	34.4 %
–Obese	29.6 %
Usual place of medicine	86.5 %

**Table 2:** Odds ratios of any healthcare visit by use of the internet to seek health information.

Variable	Model 1	Model 2	Model 3
Health information-seeking internet use	1.784***	1.673***	1.670***
Race (ref. = White)			
- Black	1.042	0.994	0.945*
- AIAN	1.121	1.021	0.876
- Asian	0.655***	0.672***	0.695***
- Multiple	0.994	0.917	0.950
Hispanic	0.828***	0.846***	0.871***
Sex (ref. = Female)	0.478***	0.465***	0.516***
Age	1.028***	1.010***	1.005***
Region (ref. = Northeast)			
- North Central	0.872***	0.842***	0.897**
- South	0.897***	0.845***	0.911**
- West	0.786***	0.770***	0.833***
Survey year	0.996	0.993	0.997
Marital status (ref. = Married)			
- Divorced/Separated	0.890***	0.895***	0.931**
- Widowed	1.052	1.050	1.106**
- Never married	0.920***	0.910***	0.968
Family income (ref.=<$50k)			
- $50-99k	1.076***	1.155***	1.081***
- $100k+	1.319***	1.499***	1.321***
Education (ref. = Less than high school)			
- High school	1.052*	1.113***	1.125***
- Some college/Associate’s	1.241***	1.315***	1.313***
- Bachelor’s degree	1.160***	1.324***	1.390***
- Beyond Bachelor’s	1.228***	1.434***	1.488***
Health insurance (ref. = None)			
- Private only	3.875***	3.825***	2.486***
- Public only	5.386***	4.409***	2.733***
- Private and public	9.589***	7.326***	4.514***
Self-rated health (ref. = “Good”)			
- Excellent		0.775***	0.765***
- Very good		0.928***	0.917***
- Fair		1.273***	1.255***
- Poor		1.949***	1.859***
BMI (ref. = Normal)			
- Underweight		0.913	0.916
- Overweight		0.998	0.987
- Obese		1.023	0.997
Chronic condition count (ref. = 0)			
- One		2.238***	2.052***
- Two or more		4.809***	4.182***
Alcohol (days of drinking per week)		0.989**	1.000
Smoking status (ref. = Never)			
- Current		0.725***	0.775***
- Former		1.138***	1.162***
- Unknown		0.868	0.840
Has a usual place of medical care			4.935***
Constant	1816.3	663793.3	343.9

* p < .05,

** p < .01,

*** p < .001.

**Table 3:** Odds ratios of any healthcare visit by frequency of internet use among users.

Variable	Model 3: All Internet Users (n = 97,045)	Model 3: Daily Internet Users (n = 79,308)
Frequency of internet use (ref. = Daily)			
- Weekly	0.791***	
- Monthly	0.884	
- Yearly	0.775	
Count of internet use per day among daily users		1.002
Race (ref. = White)		
- Black	0.943	0.956
- AIAN	0.942	1.016
- Asian	0.624***	0.613***
- Multiple	0.917	0.823*
Hispanic	0.922*	0.919
Sex (ref. = Female)	0.468***	0.450***
Age	1.004**	1.004**
Region (ref. = Northeast)		
- North Central	0.864**	0.888*
- South	0.867***	0.897*
- West	0.808***	0.845**
Survey year	0.994	0.996
Marital status (ref. = Married)		
- Divorced/Separated	0.994	0.970
- Widowed	1.088	1.068
- Never married	0.967	0.983
Family income (ref.=<$50k)		
- $50-99k	1.081*	1.079*
- $100k+	1.342***	1.330***
Education (ref. = Less than high school)		
- High school	1.198**	1.280**
- Some college/Associate’s	1.367***	1.442***
- Bachelor’s degree	1.543***	1.619***
- Beyond Bachelor’s	1.614***	1.699***
Health insurance (ref. = None)		
- Private only	2.697***	2.760***
- Public only	2.816***	2.908***
- Private and public	7.540***	8.138***
Self-rated health (ref. = “Good”)		
- Excellent	0.745***	0.750***
- Very good	0.915*	0.920*
- Fair	1.270***	1.212**
- Poor	1.819***	1.574**
BMI (ref. = Normal)		
- Underweight	0.877	0.882
- Overweight	0.955	0.950
- Obese	0.932*	0.912*
Chronic condition count (ref. = 0)		
- One	1.755***	1.732***
- Two or more	3.628***	3.680***
Alcohol (days of drinking per week)	1.007	1.016*
Smoking status (ref. = Never)		
- Current	0.784***	0.792***
- Former	1.120**	1.101*
- Unknown	1.085	0.774
Has a usual place of medical care	4.706***	4.664***
Constant	116806.1	1992.6

* p < .05,

** p < .01,

*** p < .001.

## Data Availability

NHIS data is publicly available through https://nhis.ipums.org/nhis/

## Appendix A

**Table A1:** Model 4: First and Second Differences in Age Group – Online Health Information-Seeking Interaction.

***First Differences*.** (1 = has used internet to seek health information in past year; 0 = has not)			
	Margin	Std. error	First difference
0#18-29	0.808	0.003	0.058*
1#18-29	0.866	0.002	
0#30-39	0.781	0.003	0.064*
1#30-39	0.845	0.003	
0#40-49	0.787	0.003	0.055*
1#40-49	0.843	0.004	
0#50-59	0.795	0.003	0.057*
1#50-59	0.852	0.003	
0#60-69	0.818	0.003	0.050*
1#60-69	0.868	0.004	
0#70-79	0.847	0.004	0.041*
1#70-79	0.888	0.006	
0#80+	0.858	0.005	0.035*
1#80+	0.893	0.012	

*Second Differences*.		
Age Group Comparison	Contrast (dy/dx)	Std. Error
18-29 vs. 30-39	0.006	0.005
18-29 vs. 40-49	−0.003	0.005
18-29 vs. 50-59	−0.001	0.006
18-29 vs. 60-69	−0.008	0.006
18-29 vs. 70-79	−0.017*	0.008
18-29 vs. 80+	−0.023	0.013
30-39 vs. 40-49	−0.009	0.006
30-39 vs. 50-59	−0.007	0.006
30-39 vs. 60-69	−0.014*	0.006
30-39 vs. 70-79	−0.023*	0.008
30-39 vs. 80+	−0.029*	0.013
40-49 vs. 50-59	0.001	0.006
40-49 vs. 60-69	−0.005	0.006
40-49 vs. 70-79	−0.014	0.008
40-49 vs. 80+	−0.020	−0.013
50-59 vs. 60-69	−0.007	0.006
50-59 vs. 70-79	−0.016	0.008
50-59 vs. 80+	−0.022	0.012
60-69 vs. 70-79	−0.009	0.008
60-69 vs. 80+	−0.014	0.013
70-79 vs. 80+	−0.006	0.014

**Table A2:** Model 5: First and Second Differences in Educational Attainment – Online Health Information-Seeking Interaction.

***First Differences*.** (1 = has used internet to seek health information in past year; 0 = has not)			
	Margin	Std. error	First difference
0 + less than hs	0.778	0.003	0.048*
1 + less than hs	0.826	0.006	
0 + hs grad	0.792	0.002	0.053*
1 + hs grad	0.8454	0.003	
0 + some college	0.811	0.002	0.049*
1 + some college	0.861	0.002	
0 + bachelors	0.8067	0.003	0.067*
1 + bachelors	0.873	0.002	
0 + beyond bachelors	0.819	0.005	0.057*
1 + beyond bachelors	0.8768	0.004	

***Second Differences*.**		
Contrasts (dy/dx)	Contrast	Std. error
hs grad vs. less than hs	0.006	0.007
some college vs. less than hs	0.001	0.007
bachelor’s vs. less than hs	0.019*	0.007
beyond bachelors vs. less than hs	0.009	0.008
some college vs. hs grad	−0.004	0.005
bachelors vs. hs grad	0.014*	0.005
beyond bachelors vs. hs grad	0.004	0.006
bachelors vs. some college	0.018*	0.005
beyond bachelors vs. some college	0.008	0.006
beyond bachelors vs. bachelors	−0.010	0.007

**Table A3:** Sensitivity Tests of Model 3: Ordinary Least Squares and Inclusion of Telehealth Measures (n = 500,225)

Variable	Model 3 OLS (Outcome = Count of Visits)	Model 3 + Telehealth Measures (Odds Ratios)
Health information-seeking internet use	0.427***	1.502***
Race (ref. = White)		
-Black	−0.223***	0.948*
-AIAN	−0.033	0.889
-Asian	−0.360***	0.685***
-Multiple	0.017	0.941
Hispanic	−0.159***	0.871***
Sex (ref. = Female)	−0.433***	0.531***
Age	0.002***	1.006***
Region (ref. = Northeast)		
-North Central	−0.126***	0.891**
-South	−0.053***	0.903**
-West	−0.082***	0.811***
Survey year	−0.005*	0.985**
Marital status (ref. = Married)		
-Divorced/Separated	0.018	0.926**
-Widowed	0.053***	1.114**
- Never married	0.023**	0.961
Family income (ref.=<$50k)		
- $50-99k	−0.003	1.066**
- $100k+	0.052**	1.274***
Education (ref. = Less than high school)		
- High school	0.056***	1.127***
- Some college/Associate’s	0.173***	1.230***
- Bachelor’s degree	0.241***	1.315***
- Beyond Bachelor’s	0.348***	1.370***
Health insurance (ref. = None)		
- Private only	0.541***	2.407***
- Public only	0.898***	2.727***
- Private and public	1.184***	4.520***
Self-rated health (ref. = “Good”)		
- Excellent	−0.564***	0.775***
- Very good	−0.308***	0.917***
- Fair	0.438***	1.247***
- Poor	0.939***	1.844***
BMI (ref. = Normal)		
- Underweight	0.020	0.929
- Overweight	0.002	0.988
- Obese	0.020	0.993
Chronic condition count (ref. = 0)		
- One	0.473***	2.013***
- Two or more	1.066***	4.054***
Alcohol (days of drinking per week)	−0.012***	0.996
Smoking status (ref. = Never)		
- Current	−0.077***	0.780***
- Former	0.149***	1.148***
- Unknown	−0.130	0.897
Has a usual place of medical care	0.973***	4.849***
Used internet to schedule doctor’s appointment		2.543***
Used internet to refill prescription		1.892***
